# Artificial ascites for organs at risk sparing in intrapelvic brachytherapy: a case report of recurrent uterine cervical carcinoma adjacent to the bowel

**DOI:** 10.1259/bjrcr.20180067

**Published:** 2018-08-11

**Authors:** Naoya Murakami, Satoshi Shima, Kae Okuma, Kotaro Iijima, Nikolaos Tselis, Masakazu Uematsu, Yoshiaki Takagawa, Tairo Kashihara, Koji Masui, Ken Yoshida, Kana Takahashi, Koji Inaba, Hiroshi Igaki, Yuko Nakayama, Jun Itami

**Affiliations:** 1 Department of Radiation Oncology, National Cancer Center Hospital, Tokyo, Japan; 2 Department of Radiotherapy and Oncology, Goethe-University, Frankfurt, Germany; 3 Department of Radiology, Kyoto Prefectural University of Medicine, Kyoto, Japan; 4 Department of Radiation Oncology, Osaka Medical College, Takatsuki, Osaka, Japan

## Abstract

Artificial ascites has been reported as an effective technique to reduce the risk of thermal injury in radiofrequency ablation of liver tumors by increasing the distance of collateral organs located next to the ablated sites. In this case report we share our experience with artificial ascites in an attempt to reduce the toxicity of collateral adjacent organs in the setting of re-irradiation for recurrent cervical cancer. A 52-year-old female who developed local recurrence after definitive radiation therapy was treated with interstitial re-irradiation by means of image-guided, (single-implant/multi fraction) high-dose-rate brachytherapy. Because the sigmoid colon was in close proximity to the recurrent tumor lesion, artificial ascites was generated before each treatment fraction by percutaneous injection of a defined amount of saline solution through the abdominal wall to create additional space between the two volumes. Artificial ascites showed a dosimetric improvement by reducing the sigmoid colon D_0.1cc_ per fraction from 286 cGy before to 189 cGy after saline injection. No severe complication was associated with the injection procedure.

## Introduction

Radiation therapy (RT) plays an important role in the management of uterine cervical cancer patients both as primary^1–4^ as well as postoperative adjuvant treatment.^5, 6^ However, when patients develop locally recurrent disease in pre-irradiated volumes, standard curative treatment consists of total pelvic exenteration (TPE)^7, 8^ because repeat dose-escalated external beam radiation therapy (EBRT) to the same localized site, although technically feasible, is not unreservedly implemented because of the high risk of severe side effects on account of previous RT which lower patient’s quality of life significantly.^9^ Notwithstanding, TPE *per se* constitutes a devastating surgical procedure which demands patients with both colostomy and cystostomy.^10^ Against that background, interstitial high-dose-rate (HDR) brachytherapy (BRT) has been tested as re-irradiation modality for salvage after local failure.^9, 11^ It generates biologically effective dose escalation to the treatment target whilet the versatility of intratarget dose modulation inherent to BRT can be controlled and directed to deliver higher doses to gross disease or to selectively reduce the dose to organs at risk (OARs).

From the standpoint of OARs protection, artificial ascites has been used in interventional radiology to avoid diaphragm or gastrointestinal tract damage when treating liver tumors with radiofrequency ablation (RFA).^12–15^ Factors impairing the therapeutic ratio of this thermal method are tumor size, with an accepted upper size limit of 3–4 cm for optimal treatment^16^ and the heat sink effect, stopping effective cytoreduction in perivascular lesions. In addition, there is a significant risk for thermal injury in the case of tumors abutting the diaphragm or close to the gastrointestinal tract, the bile duct, or the gallbladder. For those clinical scenarios, artificial ascites has been proven to be an effective method to increase safety space between risk structures and tumor lesions. To the best of our knowledge, there is no published experience describing the use of artificial ascites in association with RT. In the current report, we utilized it for the safe delivery of interstitial HDR BRT for the re-irradiation of recurrent cervical cancer by creating additional space between gastrointestinal tract volumes and the recurrence site.

## Clinical presentation

A 52-year-old-female was treated with definitive concurrent chemoradiotherapy for FIGO stage IIB squamous cell uterine cervical cancer. In consequence of multiple thoracic and abdominal aortic dissections for Marfan-Syndrome, radical hysterectomy was not attempted in accordance with the patient´s wish. Chemoradiotherapy consisted of 2 cycles nedaplatin (100 mg m^−^^2^) followed by 1 cycle of cisplatin (80 mg m^−^^2^) [switch from nedaplatin to cisplatin because of drug induced skin rash] and 50.4 Gy conventionally fractionated whole pelvis EBRT (central shielding after 39.6 Gy) plus 24 Gy total physical dose HDR intracavitary BRT delivered in four fractions. The patient responded with complete clinical remission.

**Figure 1.**
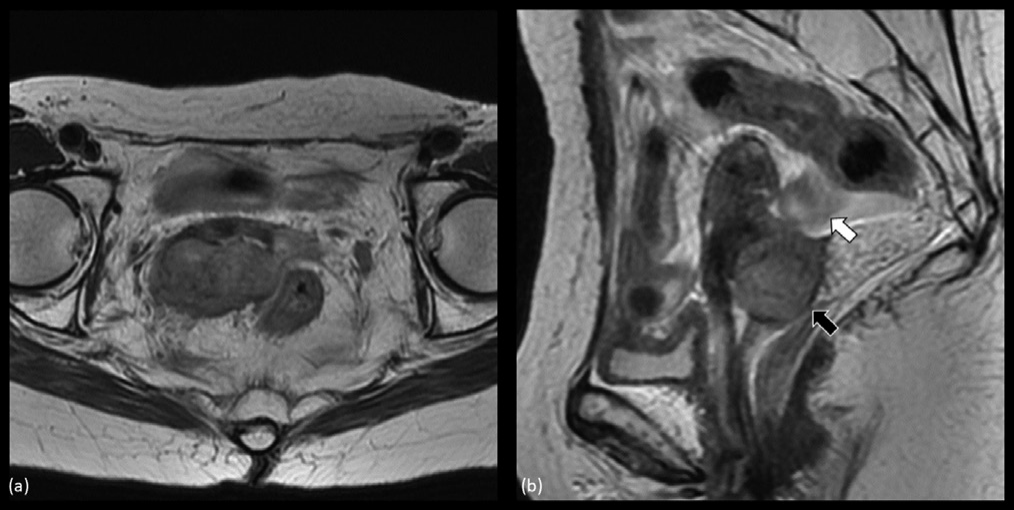
MRI of recurrent cervical cancer before salvage brachytherapy. (a) shows an axial image of the tumor. The tumor extended beyond the right-sided parametrium to the pelvic wall. (b) depicts a sagittal image of the tumor (black arrow) visualizing that the sigmoid colon (white arrow) is located just next to the recurrent lesion.

**Figure 2.**
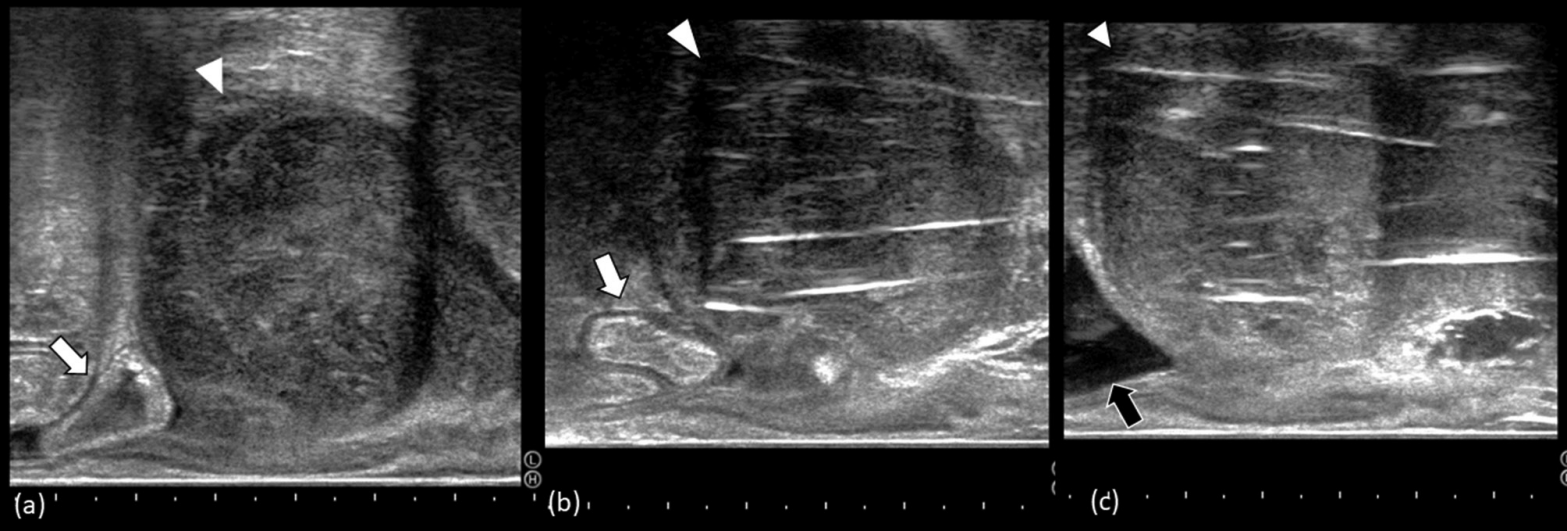
Trans-rectal ultrasonography sagittal view of the recurrent tumor. (a) shows the recurrent lesion before salvage brachytherapy. The white arrow head indicates the tumor with the white arrow marking the sigmoid colon located just next to the recurrence. b and c show the intrapelvic situs after interstitial catheter implantation. (b) depicts the situs before artificial ascites injection. It can be recognized that the sigmoid colon is situated next to the recurrent tumor. (c) is characterized by the shift of sigmoid volume outside the frame of the ultrasound (black arrow).

**Figure 3.**
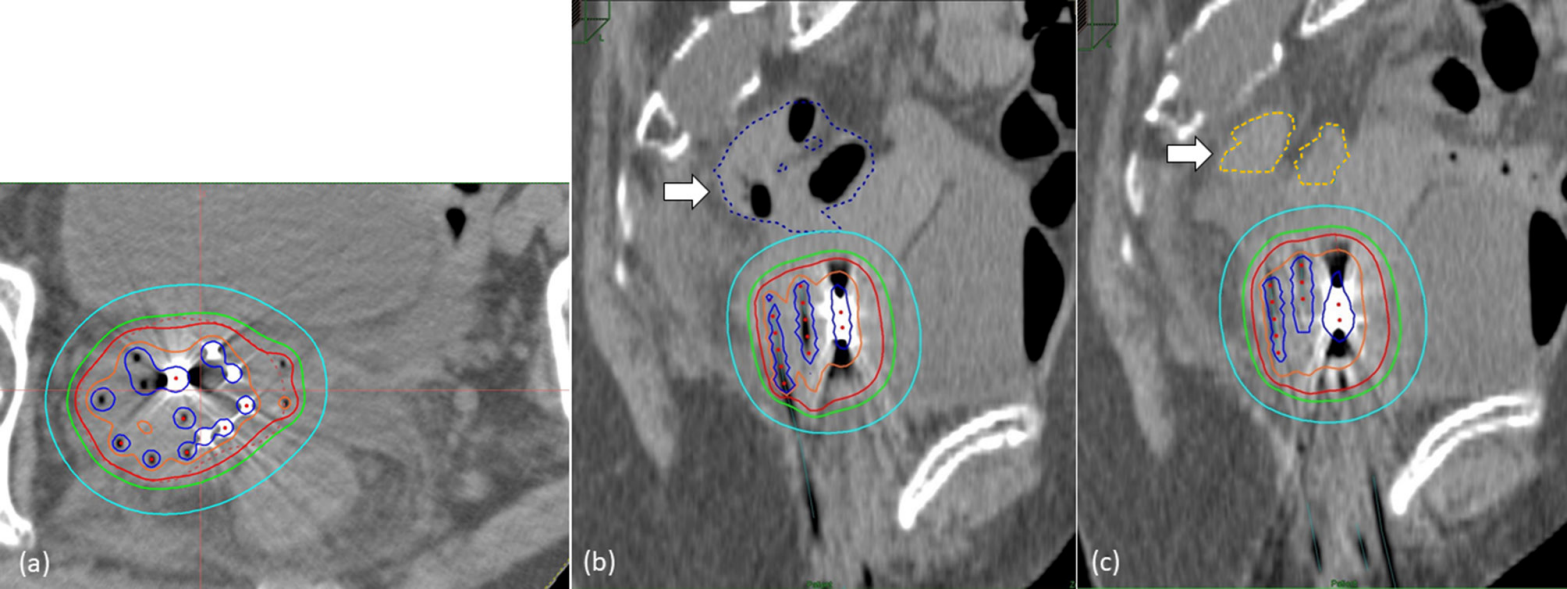
Isodose distribution of the interstitial implant. (a) demonstrates an axial view of the tumor with the red and blue line representing the 100 and 200% isodose, respectively. (b, c) depict a sagittal view before and after artificial ascites injection. It is clear that the distance between sigmoid colon and high-dose volumes is increased after artificial ascites injection.

**Figure 4.**
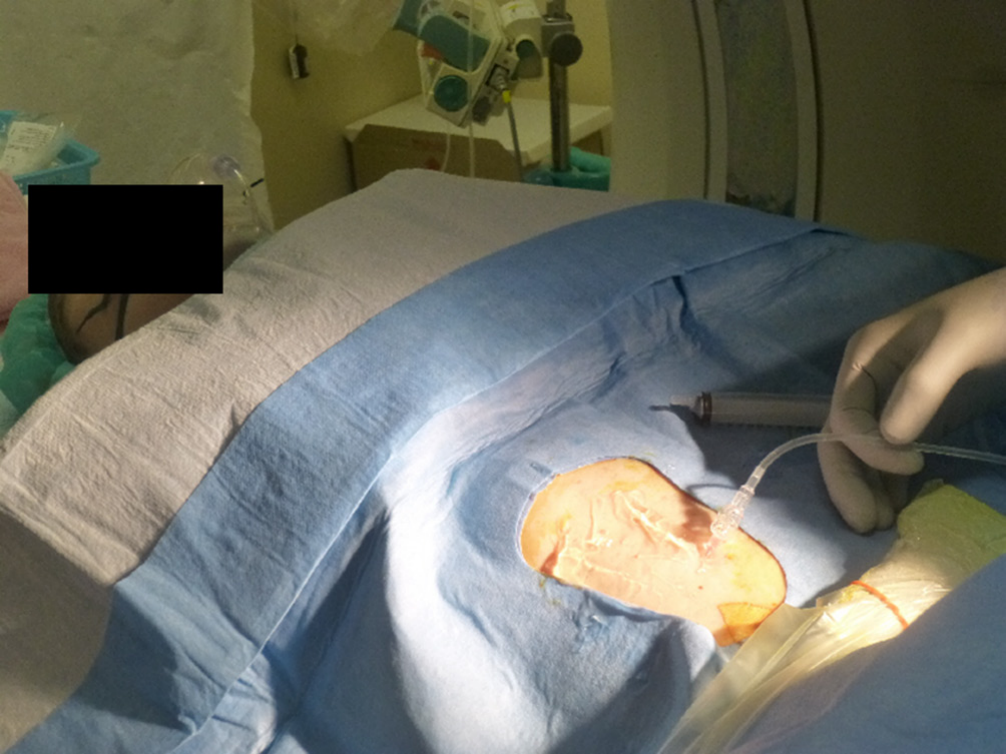
Artificial ascites injection procedure. A 20 G needle is inserted percutaneously under ultrasound guidance to avoid bowel injury and 500 ml saline solution are injected through a catheter.

One year after completion of treatment, local recurrence was detected without regional or distant metastatic disease. As first non-invasive treatment approach, 12 cycles of polychemotherapy consisting of paclitaxel (135 mg m^−^^2^) and cisplatin (50 mg m^−^^2^) followed by 6 cycles of bevacizumab (15 mg kg^−1^) was administered resulting in partial response. Considering that recurrent disease was locally confined, TPE was planned but her cardiovascular comorbidities hindered this attempt. To this end, she was referred to our department for interstitial BRT as image-guided re-irradiation method. Figure 1 shows the recurrent tumor situated in the right parametrium and extending to the pelvic sidewall. On the sagittal view, the sigmoid colon can be demarcated next to the recurrence lesion (Figure 1b). The proximity of the sigmoid colon was also clearly visualized by trans-rectal ultrasonography (TRUS) (Figure 2a). Our technique of image-guided salvage interstitial BRT for cervical cancer has been described elsewhere.^9, 11,16^ In the current case, treatment consisted of sole HDR BRT with 48 Gy total physical dose being delivered in 8 fractions at 6 Gy, applied twice-daily with an interfractional interval of at least 6 h. No additional EBRT was prescribed. Figure 3a shows the isodose dose distribution of the implant. With regard to the procedure of ascites generation, a 20G needle (Introcan^Ⓡ^ Safety, B. Braun Medical Incorporated, Bethlehem, PA) was inserted percutaneously by ultrasound-guidance and 500 ml of saline solution was administered before every BRT fraction (Figure 4). Sagittal TRUS images before and after injection are displayed in Figure 2b–c. It is shown that the injection generated a displacement of the sigmoid colon from the recurrence region, recognizable as a shift of sigmoid volume outside the frame of the ultrasound (Figure 2c). No severe complication was observed during or after the injection procedure. From a dosimetric point of view, the displacement of the sigmoid resulted in a decrease of sigmoid D_0.1cc_ from 286 to 189 cGy (Figure 3b–c) per fraction. For the purpose of the above dosimetric comparison, 3D treatment planning with anatomy-oriented dose optimization was performed before and after the first saline injection with the BRT catheters *in situ* based on repeated planning CT scans.

Written informed consent was obtained from the patient for artificial ascites injection and BRT treatment and this case report was approved by the Institutional Review Board of the National Cancer Center Hospital (approval number 2017–331) according to the ethical standards laid down in the Declaration of Helsinki.

## Discussion

Standard therapy for patients with non-metastatic recurrent cervical cancer who have past history of pelvic RT is TPE.^7, 8^ This approach poses a mutilating surgical procedure with a high incidence of complications and significant impairment of quality of life.^10^ In unresectable disease or in patients refusing surgery, radical re-irradiation with EBRT has been tried^17, 18^ but remains a choice of excessive morbidity which may outweigh the benefits of therapy. Chemotherapy alone, on the other hand, continues despite its excessive use in oncological reality to be of limited benefit with poor response and sobering outcome.^19, 20^

In this demanding clinical setting, some patients favor re-irradiation and interstitial BRT has demonstrated effectiveness in the management of locally recurrent cervical cancer developing within previously irradiated volumes.^9, 11^ There are, however, no well-defined recommendations for selecting patients for interventional radiooncological treatment and in most cases the decision is made individually. Notwithstanding this, the rationale for re-irradiation by means of HDR cannot be called into question considering that it offers radiobiological and technical advantages. As normal tissue toxicity after repeated full course conventional EBRT has shown to be significant, it seems reasonable to assume that further improvements in the therapeutic ratio can be generated by escalating the treatment dose while ameliorating conformity. Interstitial HDR BRT meets this objective optimally by exploiting the radiobiological advantage of larger fraction sizes while prospective 3D dosimetry provides anatomy-oriented dose optimization for highly conformal intensity modulated RT. At this point, the intrinsic characteristic of HDR to generate high intratarget dosing is of particular importance as it facilitates the application of ablative doses to central tumor volumes that are thought to experience increased radioresistance after previous irradiation.^21, 22^

Bearing in mind the predominantly palliative intention of re-irradiation, higher grade toxicity rates are of particular relevance the more so as a balance must be achieved between the probability of LC and the probability of toxic complications. Our group previously reported 2-year local control rates of 51.3% for patients who received image-guided interstitial BRT for recurrent cervical cancer at the cost of developing late severe complications greater than Grade 2 in 27.8% of patients.^9^ Therefore, further technical improvements are necessary to facilitate the safe delivery of cytotoxic doses. One approach could be the avoidance of hot spots in OARs by increasing the distance between target volume and OARs. In this report, we utilized artificial ascites for the safe delivery of interstitial HDR BRT of recurrent cervical cancer by creating additional space between gastrointestinal tract volumes and the recurrence site. It could be demonstrated that the procedure itself is safe and reproducible, confirming the experiences from its use in RFA of liver tumors.^11–14^ From a dosimetric point of view, the displacement of the sigmoid resulted in a decrease of sigmoid D_0.1cc_ from 286y to 189 cGy per fraction.

To the best of our knowledge, this is the first report on the feasibility and safety of artificial ascites for OARs sparing in intrapelvic BRT. Concerning its long-term efficacy, further clinical research is needed to define which kind of patients will most benefit from this technique and whether this method significantly reduces late severe adverse effects in the re-irradiation settings.

## Learning points

1. Artificial ascites has been reported as an effective technique to reduce the risk of thermal injury in radiofrequency ablation of liver tumors by increasing the distance of collateral organs located next to the ablated sites. In this case report it was suggested that a novel technique of artificial ascites in image-guided interstitial high-dose-rate brachytherapy could be generated safely in order to reduce the radiation exposure of organs at risk in the case of intrapelvic re-irradiation.
